# Complex Re-do robotic pyeloplasty using cryopreserved placental tissue: an adjunct for success

**DOI:** 10.1590/S1677-5538.IBJU.2020.0130

**Published:** 2020-11-18

**Authors:** Adam Cole, Matthew Lee, Zachary Koloff, Khurshid R. Ghani

**Affiliations:** 1 Michigan Medicine Department of Urology Ann ArborMI USA Department of Urology, Michigan Medicine, Ann Arbor, MI, USA

## Abstract

**Introduction and Objectives:**

Management of recurrent ureteropelvic junction obstruction (UPJO) following pyeloplasty presents a challenging clinical problem. Failure of initial pyeloplasty is, in part, secondary to ureteral devascularization and subsequent fibrosis. In this video, we present a case of an anastomotic augmentation with cryopreserved placental tissue (CPT) to improve tissue healing and angiogenesis, and aid with the success of re-do robotic pyeloplasty.

**Materials and Methods:**

We present a 46-year-old female with history of recurrent left-sided UPJO treated by initial endopyelotomy and then open pyeloplasty. She underwent re-do robotic pyeloplasty (DaVinci Si™, Intuitive Surgical) with CPT. The patient was placed in the flank position; a 12mm camera port, three 8mm robotic ports, and a 12mm assistant port were used. The renal pelvis and upper ureter were mobilized to reveal a dense scar at the UPJ. A dismembered pyeloplasty was performed with barbed suture. After completion of the anastomosis, a section of CPT (Stravix™, Osiris Therapeutics) was wrapped around the anastomosis. CPT is composed of umbilical amnion and Wharton's jelly, which contains a mixture of extracellular matrix, and growth factors. The CPT is prepared and thawed on the bedside table, and placed into the peritoneum through the 12mm port in the correct orientation. The wrap is secured to the anastomosis with a fibrin sealant (EVICEL™, Johnson & Johnson).

**Results:**

The patient experienced resolution of flank pain. MAG3 renogram demonstrated resolution of obstruction at 6 months, with improvement of T½ time from 34 minutes to 7 minutes, with sustained improvement with repeat scan 18 months after surgery. Ureteroscopy demonstrated a patent UPJ. Strategies for successful robotic pyeloplasty after initial failed management include: (1) use of appropriate CPT agent to support the anastomosis - selection of thicker, more durable CPT to allow passage through laparoscopic port, (2) preparation on bedside table with enough time to allow thawing, (3) marking Wharton's jelly side of tissue for orientation, and (4) use of sealing agent to secure CPT to the anastomosis and prevent dislodgement.

**Conclusions:**

We demonstrate a novel approach to manage recurrent UPJ obstruction with robotic surgery using CPT. Placenta-derived products may have an increasing role in the performance of complex robotic urologic reconstructive surgery.
